# Residual foci of *Triatoma infestans* infestation: Surveillance and control in Rio Grande do Sul, Brazil, 2001-2018

**DOI:** 10.1590/0037-8682-0530-2020

**Published:** 2021-03-08

**Authors:** Cleonara Bedin, Tânia Wilhelms, Marcos Marreiro Villela, Guilherme Carlos Castilhos da Silva, Ana Paula Konzen Riffel, Paulo Sackis, Fernanda de Mello

**Affiliations:** 1 Secretaria Estadual da Saúde, Centro Estadual de Vigilância em Saúde, Porto Alegre, RS, Brasil.; 2 Universidade Federal de Pelotas, Departamento de Parasitologia, Pelotas, RS, Brasil.; 3 Secretaria Estadual da Saúde, Coordenadoria Regional de Saúde de Santa Rosa, Santa Rosa, RS, Brasil.; 4 Secretaria Estadual da Saúde, Centro Estadual de Vigilância em Saúde, Laboratório Central de Saúde Pública, Porto Alegre, RS, Brasil.

**Keywords:** Triatoma infestans, Chagas disease, Entomological surveillance, Vector control

## Abstract

**INTRODUCTION:**

This retrospective study conducted from 2001 to 2018 investigated the residual foci of *Triatoma infestans* infestation in Rio Grande do Sul, Brazil.

**METHODS:**

The data were obtained via entomological surveillance and the distribution of vector occurrence. The coverage of active research was mapped.

**RESULTS:**

The largest coverage rate for active research was observed in the northwest region of the total of 515,081 domiciles researched. Most *T. infestans* specimens were captured in the peridomicile.

**CONCLUSIONS:**

Infestation has decreased significantly since 2008, and *T. infestans* has not been captured since 2015.

*Triatoma infestans* was the main insect vector of the protozoan *Trypanosoma cruzi*, an etiologic agent of Chagas disease (ChD), one of the most important parasitic diseases in Latin America^1^. In 1991, it was indicated that the control of *T. infestans* in endemic risk areas should be the focus of a collaborative effort by the Ministries of Health in the Southern Cone Initiative: Argentina, Bolivia, Brazil, Chile, Paraguay, and Uruguay. The Pan American Health Organization/World Health Organization (PAHO/WHO) established and coordinated the "Intergovernmental Commission on Chagas Disease," which prepared the "*Triatoma infestans* Elimination Plan" for the elimination of *T. infestans* and the interruption of transfusion transmission of trypanosomiasis. The plan defined vector elimination as follows: no capture of the vector for a minimum period of 3 years in an area with established and functional entomological surveillance^2^. The elimination plan was successful. There was an 83% reduction in the occurrence of *T. infestans* in Brazil in 1993, with some residual foci remaining in the states of Rio Grande do Sul (RS) and Bahia^3^.

This study aimed to evaluate the elimination of the vector and long-term entomological surveillance in RS. RS is located in south Brazil. RS shares a northern border with the State of Santa Catarina and international boundaries with Uruguay to the south and southwest, and Argentina to the west and northwest. RS has a humid subtropical climate and a variety of temperatures throughout the year, recording sub-zero temperatures in the winter and temperatures near 40 °C in the summer.

Entomological surveillance of ChD was developed by the state through the Chagas Disease Control Program (ChDCP) under the State Health Surveillance Center (Centro Estadual de Vigilância em Saúde [CEVS]) and the State Department of Health (SES-RS). The actions were developed by 19 Regional Health Coordinators (Coordenadorias Regionais de Saúde [CRS]) and 497 municipal health departments. Entomological data sets were obtained from the information system ChDCP-DATASUS-MS (Information Technology Department) between 2001 and 2016, and the 2017-2018 entomological data sets were obtained from the form-generation system of the Unified Health System (FormSUS)- DATASUS-MS. 

The insects were collected from Passive Surveillance (PS) through the Triatomine Information Post (TIP), also known as community surveillance (notified by the population); Active Research (AR); and integral research (IR), the active search of vectors in all of a positive locality for *T. infestans* in the previous year, and the active search in nearby localities. 

The AR followed the field operations planning protocols in the rural areas. Public health agents were trained for AR and received supervision during fieldwork. Triatomine research was carried out using tweezers, an insect dislodging product, and a flashlight to better see the triatomines' hiding places in the intradomicile and peridomicile environments. However, it is important to report that capture failures are likely to occur, both on the part of the population and health workers. Therefore, continuous refreshers and training courses and educational programs must be provided. 

The dataset included the following: year; number of municipalities with *T. infestans* infestation; positive domicile units (DUs) with *T. infestans* infestation and ecotope of capture in the DU occurrence of the capture in intradomicile (ID) and/or peridomicile (PD) areas; capture of 1 or > 1 insect; the presence of nymphs (N) in the ID and/or PD areas (nymphs characterize colonization of the DU); the total number of DUs researched; the total number of sprayed DUs; number and productivity of TIPs for triatomines; and other insects captured in the year (Table 1).

**TABLE 1:** Number of municipalities and domicile units by ecotope of *Triatoma infestans* capture and entomological surveillance and control activities in Rio Grande do Sul, Brazil (2001-2018).

*Triatoma infestans*											Surveillance and Control Activities				
Year	Mun. +	Intradomicile				Peridomicile				Total	DUs	** DUs	TIPs	Triat.	Other
		*+	1	>1	N	+	1	>01	N	DUs+	researched	sprayed			Insects
2001	14	16	11	5	4	20	10	10	10	36	40644	3420	2293	1071	8
2002	16	16	14	2	3	11	3	8	7	27	35329	3845	2100	885	6
2003	13	6	4	2	4	24	7	17	14	30	65584	1788	1938	1183	49
2004	13	6	3	3	0	10	3	7	6	16	70899	902	2206	1262	20
2005	15	10	7	3	2	17	4	13	9	27	65183	795	2241	1540	167
2006	9	6	6	0	0	12	5	7	6	18	54474	975	2313	1570	239
2007	11	4	2	2	2	11	1	10	7	15	37674	451	2245	1081	114
2008	4	3	1	2	1	2	1	1	1	5	37959	374	2282	795	93
2009	2	0	0	0	0	2	1	1	1	2	28482	441	2261	696	89
2010	2	2	2	0	0	0	0	0	0	2	16667	290	2260	445	167
2011	0	0	0	0	0	0	0	0	0	0	32866	355	2239	589	206
2012	1	2	2	0	2	1	1	0	0	3	11645	59	2146	604	445
2013	2	1	0	1	1	1	0	1	1	2	4331	31	2019	337	521
2014	1	0	0	0	0	5	1	4	3	5	4180	32	2164	376	610
2015	0	0	0	0	0	0	0	0	0	0	3328	13	2143	220	679
2016	0	0	0	0	0	0	0	0	0	0	2575	10	2109	177	396
2017	0	0	0	0	0	0	0	0	0	0	2536	2	2117	229	1579
2018	0	0	0	0	0	0	0	0	0	0	725	4	1988	208	1555
**Total**		**72**	**52**	**20**	**19**	**116**	**37**	**79**	**55**	**188**	**515081**	**13787**	**-**	**13268**	**6943**

*+: No. of positive DUs; 1: foci with one *T*. *infestans*; > 1: foci with more than 1 *T*. *infestans*; N: DUs with nymphs; **Deltamethrin pyrethroid. Triatomines; TIP: Triatomines Information Post; Other Insects: arthropods collected.

The area of vector occurrence was mapped, overlapping the area of AR, with the software QGIS. The intensity of vector occurrence was established by the number of years during which *T. infestans* were identified in the municipalities between 2001 and 2018. These data were categorized by capture frequency: 1 year, 2-4 years, 5-6 years, and > 6 years. AR was evaluated using the coverage rate of AR (AR%). The AR% by year was defined as the number of DUs found by ChDCP divided by the number of rural DUs, from 497 municipalities in RS, by year, over 18 years. The AR% (total average) is the result of the division of the total accumulated coverage rate of active research by year and municipality, over 18 years (2001-2018). The AR% was classified as follows: < 1%, > 1% to 15%, > 15% to 30%, > 30% to 45%, and > 45% to 65%. Municipalities (eight) with little or no rural population were categorized as < 1. The AR% was mapped and overlapped with the vector occurrence in the RS territory between 2001 and 2018. The *T*. *infestans* frequency from 1 to 6 years occurred in 19 municipalities (Figure 1).

**FIGURE 1:**
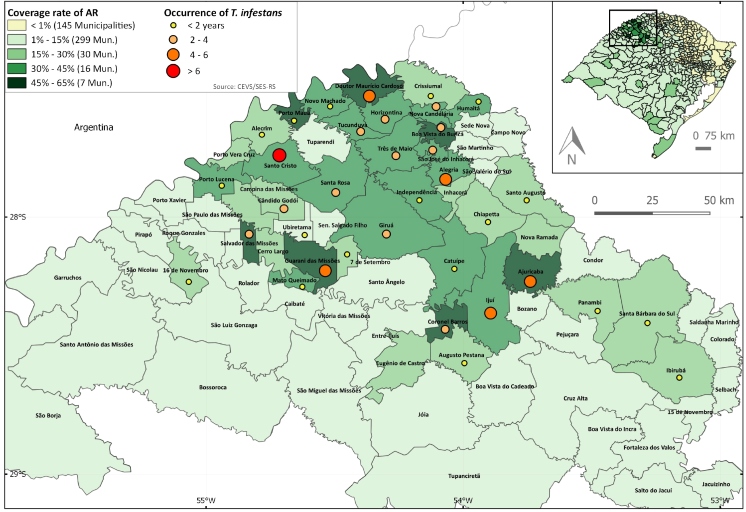
Coverage rate of active research and occurrence of*Triatoma infestans*capture in the Northwest region of Rio Grande do Sul, Brazil, from 2001 to 2018.

The distribution of the last foci of *T. infestans* infestation from 2012 to 2014 was restricted to 3 municipalities (Figure 2). The PS (notified by the population) was motivated by the installation and maintenance of the TIP and educational activities for health promotion. An awareness campaign was launched in 2012 with promotional materials such as radio spots, posters, and brochures, reprinted and used in subsequent years^4^. The ChDCP promoted > 150 events or meetings between 2011 and 2017. Additionally, the Universidade Federal de Pelotas, in partnership with the SES-RS and the Telessaúde-RS (UFRGS), produced educational materials for ChD prevention activities: the film “Chagas Disease Documentary,” available for free on DVD and with free internet access^5^; and an environmental surveillance calendar for 2017 with facts about ChD vectors and their control. 

**FIGURE 2:**
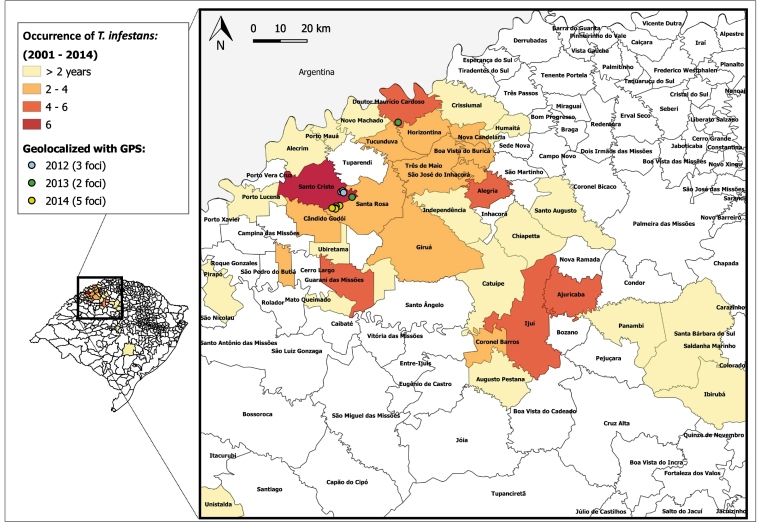
Distribution of the occurrence of *Triatoma infestans*capture from 2001 to 2014, and the geolocation of the last foci in 2012-2014 in Rio Grande do Sul, Brazil. *2011 no foci.

The number of AR and sprayed DUs decreased in the historical series. AR was highest between 2001 and 2005, as a result of the surveillance efforts in the municipalities with *T. infestans* residues for the control of ChD to obtain a Certificate of Interruption of Transmission of Chagas Disease by *Triatoma infestans*.

Greater AR coverage occurred in the region considered high risk for *T. infestans,* since all municipalities reported the occurrence of the vector in the past. These municipalities had foci of *T. infestans* between 1 and 6 years. The municipalities’ areas were scheduled for AR and IR, and an AR% of 45-65% was found in 7 municipalities. The number of AR decreased in 2012 because of the consistent drop in the number of triatomines captured by health agents in rural DU. Active surveillance and spraying carried out by the health agents proved to be effective in the presented scenario within the areas with the occurrence of *T*. *infestans*.

Most *T. infestans* specimens were captured in PD areas, and the presence of more than one specimen was also more likely in PD areas than in ID areas. The number of colonies (the presence of nymphs in the ecotope) was also higher in the PD environment. Infestations with one *T. infestans* vector were more common in ID areas. With respect to the invasion site, the infestation of DUs was consistently more common in PD areas than in ID areas, similar to results verified in Argentina^6^.

The foci were treated with residual action insecticide and integrated into the FUNASA's Housing Improvement Program for Chagas Disease Control (HIPCDC). Additionally, educational activities were implemented, as recommended by the Brazilian Consensus on Chagas disease. For housing improvement, the SES-RS allocated financial resources, based on presence of *T. infestans*, to the HIPCDC for the construction and renovation of DUs with outhouses in rural localities in northwest RS, making these areas inhospitable to triatomine colonization^7^. It is important to note that most improvements occurred in the peridomicile areas, including painting and renovations. Moreover, the teams intensified their work in hen houses and storerooms to prevent the entry of animals that could serve as *T*. *cruzi* reservoirs or triatomine food sources. This process triggered a series of trainings, domicile research, educational workshops, and the administrative and technical reorganization of ChDCP. Indeed, this contributed to the disappearance of the residual foci of *T*. *infestans* infestation, found after the implementation of the HIPCDC^8^.

With respect to the consistent decrease in the number of triatomines, the entomologic scenario indicated other directions for the program: a major investment in the passive or community surveillance, with the installation and maintenance of TIPs in all municipalities of the state with the collected insects. Community participation in ChD vector surveillance is fundamental to the success of entomological control and, with community involvement, surveillance becomes continuous and is not performed by only ChDCP agents, resulting in long-term, sustainable control^9^ ^,^ ^10^. The number of reports of triatomines decreased during this period, but the productivity of TIPs has increased, as observed by the increase in the total number of arthropods in recent years, demonstrating that the educational actions carried out with the disclosure of TIPs have achieved the intended pedagogical effect.

The causes that may have influenced the occurrence and maintenance of populations of *T. infestans* in the northwestern region of RS, especially in the first years of the investigation, are as follows: climate of the region, since northwest RS has warmer annual average temperatures of 20-22°C (higher than that in other areas of the state: variations between 15 and 18°C, and a minimum average of 10ºC in the winter); socio-cultural profile of individuals from the northwest region (mainly Italian and German immigrants); and several outbuildings (storerooms, sheds, chicken houses, and brick ovens) and the accumulation of deposits and building materials (woods and bricks) in rural DUs in the northwest, which can lead to failures in spraying activities. These factors are known to favor infestation and might explain the recolonization^11^ ^,^ ^12^. 

It is presumed that there was operational failure in the actions of the Mobilization Project for the Elimination^13^ of *T*. *infestans* implemented in 2010 in the municipality of Santa Rosa (Figure 2), since five foci of the vectors were found in 2014. It is suspected that the reported focus of the Church in Santo Cristo (2012-2013), a town near Santa Rosa, may have caused the spread of *T*. *infestans* in the region.

With respect to spraying, it has been reported that insect resistance to the chemical treatment employed (pyrethroid insecticides such as alpha-cypermethrin and deltamethrin) was not observed. In addition, Pessoa et al. (2015)^14^ and Belisário et al. (2017)^15^ demonstrated that the specimens of *T*. *infestans* strong genetic structure and little to no gene flow among populations, from the states of Bahia and RS, collected precisely in Santa Rosa and Doutor Maurício Cardoso. These municipalities, together with Santo Cristo, exhibited the last foci of *T*. *infestans* in this investigation and were susceptible to pyrethroid insecticide deltamethrin in laboratory tests, corroborating our field observations. 

The infestation has decreased significantly since 2008, and *T*. *infestans* was not captured in 2015, 2016, 2017, or 2018. The last foci were recorded in 2014. Thus, it is essential to note that the persistent occurrence of *T. infestans* in RS did not occur due to the entry of triatomines from other sources, including neighboring countries, and that the residual infestations cannot be attributed to resistance to pyrethroids.

After achieving the vector elimination plan, the main issue was to maintain the structure and provide surveillance coverage avoiding the reemergence of ChD. Thus, it was emphasized that entomological surveillance should be permanent, with the participation of health services and the community. These are points of vital importance to achieve sustainable control. The operational routine maintenance of AR in in areas without the domiciled vector is justifiable in the public administration only if combined with a vision of environmental health surveillance, including educational activities and the addition of a housing improvement component to ChD control, mandating environmental sanitation and care in rural housing. Although it is too early to determine whether long-term control will be achieved, the results confirm the efforts in the search to eliminate *T. infestans* in RS, coordinated by SES, municipal departments, public health agents, and the community.
